# Supplementary material for: Functional and Structural Changes in Diaphragm Neuromuscular Junctions in Early Aging
Source: Int J Mol Sci. 2024 Aug 17;25(16):8959. doi: 10.3390/ijms25168959 (PMC11354816; doi:10.3390/ijms25168959)
Supplement: Supplementary file 1 [file ijms-25-08959-s001.zip › ijms-3148282-supplementary.pdf]

S. Table S1. Comparison of EPP and MEPP parameters in 3- vs 9-month-old mice

Parameter	3 months (n=64-68)	9 months (n=14)	Significance of difference
Latency	1.184±0.242 ms	1.140±0.167 ms	P=0.37
Synaptic delay	0.598±0.114 ms	0.535±0.107 ms	P=0.14
Latency including synaptic delay	1.824±0.364 ms	1.727±0.178 ms	P=0.33
EPP Amplitude	7.53±1.72 mV	6.66±2.27 mV	P=0.11
EPP rise time	0.242±0.049 ms	0.230±0.057 ms	P=0.41
EPP decay time	1.634±0.241 ms	1.506±0.157 ms	P=0.06
Quantal content (m)	21.40±6.22	19.35±5.02	P=0.25
Rt/rt	1.44±0.22	1.47±0.32	P=0.61
Frequency of MEPPs	0.93±0.43 s^{-1}	1.07±0.47 s^{-1}	P=0.26
MEPP amplitude	0.37±0.12 mV	0.36±0.17 mV	P=0.80
MEPP rise time	0.167±0.020 ms	0.156±0.015 ms	P=0.06
MEPP decay time	1.386±0.172 ms	1.329±0.099 ms	P=0.25

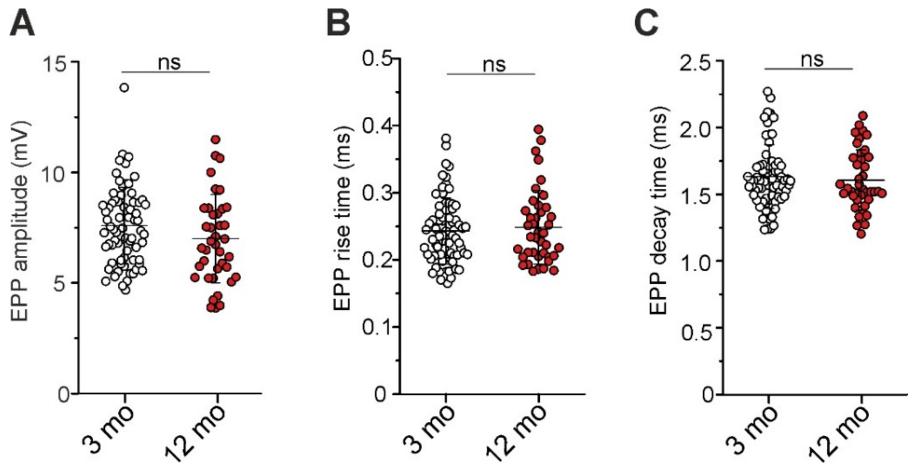

S. Figure S1. Parameters of evoked postsynaptic responses. Mean values of amplitude**(A)**, rise time **(B)** and decay time **(C)** of EPPs evoked at 0.5 Hz stimulation are shown. n = 70 and 40 for 3 and 12 months. ns -non significant.

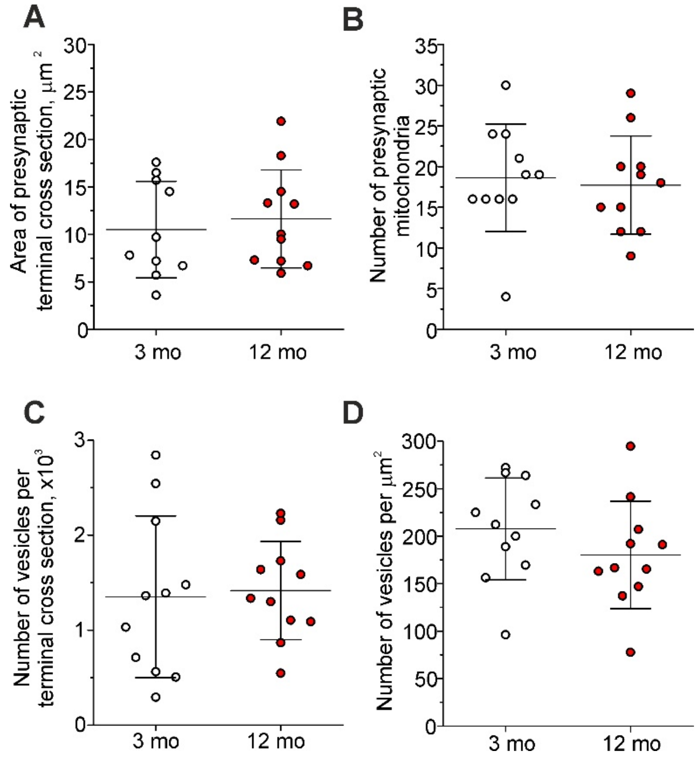

S. Figure S2. Morphology of presynaptic nerve terminals. Graphs show area of presynaptic nerve terminal cross section (**A**), number of mitochondria in cross section of presynaptic terminal (**B**), number of vesicles per nerve terminal cross section (**C**), number of vesicles per mm² of nerve terminal cross section. Quantifications were made based on electron microscopy images. n=3 mice and 10-11 presynaptic nerve terminal cross sections per group.
